# Emerging Regenerative Medicine for Spinal Cord Injury: Spinal Cord Organoids-on-a-Chip

**DOI:** 10.3390/ijms27157060

**Published:** 2026-08-06

**Authors:** Manzar Khan, Hyunjin Choi, Sareer Ahmad, Somin Lee, Jong-Chan Park, Inbo Han

**Affiliations:** 1 Department of Biomedical Science, CHA University, Seongnam-si 13488, Republic of Korea; manzarkhan07@gmail.com (M.K.); sareerkhan2020@gmail.com (S.A.); 2 Department of MetaBioHealth, Sungkyunkwan University, Suwon 16419, Republic of Korea; hjchoi0@skku.edu; 3 Department of Neurosurgery, CHA Bundang Medical Center, CHA University, Seongnam-si 13496, Republic of Korea; dmddmdmd5@gmail.com; 4 Department of Biophysics, Institute of Quantum Biophysics, Sungkyunkwan University, Suwon 16419, Republic of Korea; 5 Department of Biopharmaceutical Convergence, Sungkyunkwan University, Suwon 16419, Republic of Korea

**Keywords:** spinal cord organoids, microfluidic platform, spinal cord injury, biomimetic platform, regenerative medicine, organoid-on-a-chip

## Abstract

Spinal cord injury (SCI) is among the most devastating neurological conditions worldwide, imposing substantial clinical, psychological, and socioeconomic burdens on affected individuals and healthcare services. However, a translational gap persists between preclinical findings and therapeutically relevant human applications, partly involving the limitations of conventional two-dimensional culture systems, which inadequately reproduce the complex three-dimensional structure of the spinal cord; and partly due to the fact that animal models differ from humans phylogenetically and physiologically. Recent advances in three-dimensional spinal cord organoids and organoid-on-a-chip microfluidic platforms have provided new opportunities to model human spinal cord biology with greater fidelity. This review discusses the clinical and socioeconomic burden of SCI, identifies the limitations of existing experimental platforms, and explains how microfluidics and scaffold technologies may converge to accelerate the development of regenerative therapies. It also summarizes methodological innovations, recent advances, unresolved challenges, and future prospects in this rapidly evolving field.

## 1. Introduction

Spinal cord injury (SCI) is a serious neurological condition that can cause permanent motor, sensory, and autonomic impairment [1]. SCI is most commonly traumatic, resulting from mechanisms such as sports injuries, traffic accidents, falls, and violence, although non-traumatic causes include infection, tumors, and spinal degeneration. According to the World Health Organization (WHO), the global annual incidence of SCI ranges from 10 to 83 cases per million population, with approximately 90% of cases caused by trauma and 10% caused by non-traumatic etiologies [2,3,4]. The socioeconomic burden of SCI is substantial; in high-income countries, lifetime medical care costs are estimated at approximately USD 2–5 million per patient, including productivity losses and caregiving costs [5]. Although acute trauma care and rehabilitation have improved, approximately 30% of people with SCI experience severe lifelong disability accompanied by secondary pathologies, including neuropathic pain, urinary and respiratory tract infections, and cardiovascular dysfunction [6,7]. Current standard care remains largely supportive, and no therapy has yet achieved meaningful functional restoration, underscoring the urgent need for regenerative medicine approaches [8,9].

Traditionally, two-dimensional (2D) monolayer cultures have been widely used as foundational in vitro models in SCI research, but they have important limitations. Cell monocultures grown in rigid 2D microenvironments cannot recreate the three-dimensional (3D) architecture, extracellular matrix (ECM) interactions, multicellular complexity, or dynamic microenvironmental cues, such as fluid flow, oxygen gradients, and mechanical stimulation, that regulate spinal cord biology in vivo [10,11]. Importantly, the blood–spinal cord barrier (BSCB), a key pathological feature of SCI and a major determinant of drug permeability into the spinal cord, cannot be adequately reproduced in conventional 2D culture systems [12]. In vivo rodent models have provided fundamental mechanistic insights, but a substantial translational gap persists between preclinical findings and clinical outcomes. Rodents differ substantially from humans in corticospinal tract organization, white-to-gray matter ratio, spontaneous regenerative capacity, and neuroinflammatory gene-expression profiles. This limitation is reflected in several high-profile clinical failures, including methylprednisolone, riluzole, minocycline, Cethrin, and anti-Nogo antibody therapies [13,14]. Although large-animal models, such as pigs and non-human primates, offer greater anatomical and physiological relevance, their use is constrained by high costs, ethical concerns, and limited genetic tractability. Together, these limitations, along with the ethical principles of the 3Rs paradigm (replacement, reduction, and refinement), have driven the development of more physiologically relevant human in vitro platforms for SCI research [15].

Spinal cord organoids (SCOs) are 3D self-organizing structures derived from human embryonic stem cells (hESCs) or human induced pluripotent stem cells (hiPSCs), and they have emerged as promising models of human spinal cord biology. SCOs can recreate key features of cellular diversity, cytoarchitectural organization, and electrophysiological function [16]. Mechanistically, sequential neural induction through dual SMAD inhibition, followed by caudalization through Wnt/fibroblast growth factor 2 (FGF-2) signaling and dorsoventral patterning through sonic hedgehog (SHH) or bone morphogenetic protein 4 (BMP4), produces organoids containing motor neurons (ISL1^+^, HB9^+^), interneurons, astrocytes, and oligodendrocyte precursors (OLIG^+^) [17,18]. SCOs also exhibit functional electrophysiological properties, including spontaneous action potentials, synaptic transmission, and oscillatory network activity, which can be recorded using microelectrode arrays (MEAs) and calcium imaging. In addition, single-cell RNA sequencing analyses have shown transcriptomic convergence with primary human spinal cord tissue, supporting the use of SCOs as emerging human-specific disease models [19].

Organoid-on-a-chip (OoaC) platforms complement organoids by enabling precise control of the cellular microenvironment within microfabricated fluidic channels. These systems combine the self-organizing properties of organoids with controlled biochemical, mechanical, and fluidic parameters by embedding stem cell-derived organoids in engineered microfluidic devices [17,20]. Huh et al. (2010) previously demonstrated that organ-on-a-chip (OoC) platforms can integrate controlled fluid flow, biomechanical stimulation, oxygen and nutrient gradients, and compartmentalized co-culture to recreate organ-level physiology [21]. Therefore, OoaC platforms incorporating stem cells, astrocytes, and endothelial cells have been developed to model SCI, the BSCB, and neuroinflammation, while integrated biosensors enable longitudinal functional monitoring [16]. These systems may also support nutrient delivery across diffusion barriers, regulate flow dynamics, incorporate vascular networks, establish biochemical gradients that model the injury penumbra, and enable real-time functional assessment.

This review discusses how integrating SCOs with microfluidic OoaC platforms might provide a human-relevant experimental approach for modeling SCI pathobiology and testing neuroprotective and regenerative strategies, including cell therapies, biomaterial scaffolds, neurotrophic factors, and pharmacological agents. By combining insights from developmental biology, bioengineering, neuroscience, and clinical medicine, this review outlines a framework for accelerating therapeutic development in SCI. Unlike previous reviews, the present review provides a comprehensive and translational perspective on SCO-OoaC technology by critically integrating advances in stem cell biology, biomaterials, and microengineering for SCI research. The functional integration of spinal cord-specific ECM scaffolds with human iPSC-derived spinal cord organoids is an important focus of this review, showcasing the potential of tissue-specific ECM scaffolds to enhance organoid maturation, biomimicry, and regenerative capacity in microphysiological platforms. In addition to addressing the key biological and engineering challenges associated with SCO-OoaC development, this review proposes a streamlined translational roadmap to facilitate the clinical and preclinical implementation of SCI-specific SCO-OoaC systems. In addition, a systematic literature search was conducted through PubMed, Google Scholar and ResearchGate, as well as other relevant scientific sources, focusing specifically on publications between 2010 and 2025. Moreover, this review is a novel combination of new developments in microfluidic morphogen patterning, microelectrode array (MEA) electrophysiology analysis, and spinal cord-relevant ECM engineering in SCO-OoaC platforms. To provide a comprehensive technological framework, fundamental studies of brain organoids, neural tube models and BSCB/BBB-on-a-chip system are also discussed when the methodology is directly applicable and translatable to the SC on-a-chip models. This review brings together these multidisciplinary advances in a single SCI-focused framework to highlight current gaps in knowledge, emerging engineering strategies, and directions for advancing physiologically relevant and clinically translatable SCO-OoaC platforms for regenerative medicine.

## 2. Cellular and Molecular Mechanisms of SCI

### 2.1. Biphasic Pathophysiological Mechanisms: Primary and Secondary Injury

The pathophysiology of SCI is characterized by a stereotypical biphasic cascade. Primary mechanical trauma, including contusion, compression, laceration, or distraction of neural tissue, immediately disrupts neural tissue architecture, microvasculature, and cell membrane integrity at the injury epicenter [22]. These initial traumatic events establish the lesion volume and are largely irreversible and generally refractory to pharmacological intervention. Ionic dysregulation also occurs immediately after primary injury and is characterized by uncontrolled Ca^2+^ influx through voltage-gated and mechanosensitive ion channels, Na^+^ overload mediated by persistent activation of voltage-gated sodium (Nav) channels, and K^+^ efflux leading to membrane depolarization [23,24]. This ionic imbalance subsequently initiates excitotoxicity through excessive glutamate-mediated activation of N-methyl-D-aspartate (NMDA) and α-amino-3-hydroxy-5-methyl-4-isoxazolepropionic acid (AMPA) receptors. Intracellular Ca^2+^ accumulation further activates calpain-mediated cytoskeletal proteolysis and phospholipase activity, resulting in lipid peroxidation, mitochondrial dysfunction, oxidative stress, and progressive cellular damage [25,26].

**Figure 1:**
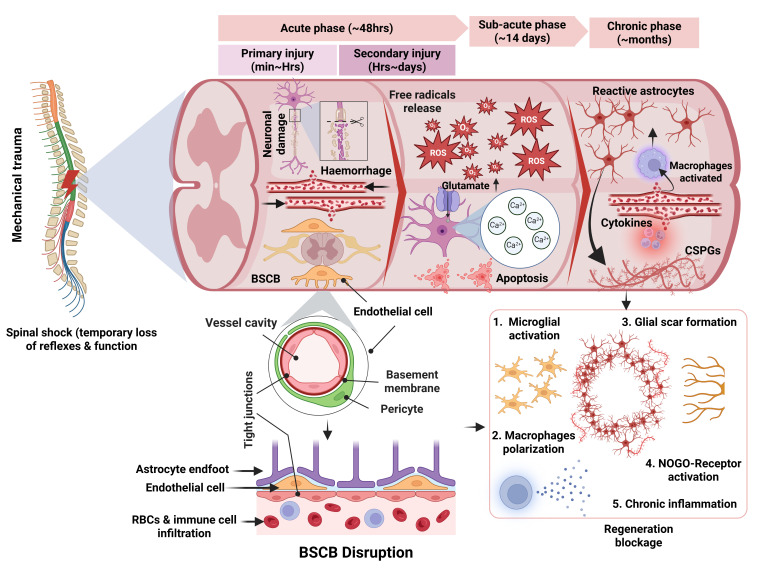
**Pathophysiological progression of SCI and BSCB disruption.** Schematic representation of the acute (~48 h), subacute (~14 days), and chronic (~months) phases of SCI. Primary injury refers to mechanical tissue damage, hemorrhage, and BSCB disruption, whereas secondary injury involves reactive oxygen species (ROS) generation, glutamate excitotoxicity, Ca^2+^ overload, apoptosis, and neuroinflammation. Activated macrophages, reactive astrocytes, inflammatory cytokines, and CSPGs contribute to glial scar formation and inhibition of axonal regeneration. The lower panel shows the structural elements of the BSCB: endothelial cells, tight junctions, basement membrane, and pericytes. Created in BioRender. Roh, E. (2026) https://BioRender.com/5tk4up8 (Agreement number: AY29TP9RDS).

BSCB disruption occurs within minutes after primary injury (Figure 1). This disruption is mediated by matrix metalloproteinase 2 (MMP-2) and matrix metalloproteinase 9 (MMP-9), which target tight-junction proteins, including claudin-5, occludin, and ZO-1, thereby permitting early neutrophil infiltration, which peaks at 12–24 h after injury, and the accumulation of CCR2+ monocyte-derived macrophages [27,28]. The subacute stage, from days to weeks after injury, is marked by continued oligodendrocyte apoptosis and demyelination of structurally intact axons remote from the primary lesion; this potentially reversible process is a key target of remyelination strategies and contributes to the development of a tripartite lesion architecture. The chronic stage, from weeks to years after injury, is characterized by glial scar consolidation, cystic cavity formation, Wallerian degeneration of disconnected axonal tracts, and maladaptive sequelae, including neuropathic pain, spasticity, and autonomic dysreflexia, which substantially influence long-term functional outcomes and quality of life [29,30].

### 2.2. Cellular and Molecular Architecture of Glial Scar and Neuroinflammation

#### 2.2.1. Multicompartmental Scar and Reactive Astrogliosis

Post-SCI lesions are characterized by tricompartmental scar development, which has been systematically studied using spatial transcriptomics. These analyses identified 21 transcriptional cell states with specific spatial distributions across the injury epicenter and perilesional tissue [31]. The lesion contains an inner fibrotic core composed of pericyte-derived fibroblasts, macrophages, and lymphocytes embedded in a collagen- and fibronectin-rich matrix; an intermediate astroglial border composed of proliferating reactive astrocytes (GFAP^+^, vimentin^+^, CXCL10^+^) and NG2^+^ oligodendrocyte precursor cells (OPCs); and inhibitory proteoglycan-secreting cellular components. Although the astroglial border is acutely neuroprotective by mechanically confining inflammation and limiting lesion expansion, its chronic persistence creates a regenerative barrier through dense chondroitin sulfate proteoglycan (CSPG) deposition that suppresses axonal regrowth, physical exclusion of axonal growth cones from the lesion core, regulation of pro-scar gene networks by STAT3, and recruitment of additional scar-forming astrocytes [31,32].

#### 2.2.2. Neuroinflammatory Cellular Responses and Inhibitory Post-SCI ECM

Microglial polarization dynamics play a central role in determining the extent of secondary injury and the regenerative capacity of the post-SCI niche. During the first week after injury, M1-like (iNOS^+^, CD86^+^) microglia and infiltrating macrophages predominate (Figure 1), releasing a proinflammatory cytokine milieu that includes TNF-α, IL-1β, IL-6, IL-12, and reactive oxygen species, thereby aggravating neuronal apoptosis and oligodendrocyte death. A transient M2-like (Arg1^+^, CD206^+^, IL-10^+^) anti-inflammatory phase is insufficient to establish sustained inflammatory resolution [33,34]. A recent single-cell RNA sequencing study revealed a “second wave” of Hif1α-driven microglial repopulation that dominates the microglial landscape at the injury site by day 14 after SCI and permanently channels these cells into a disease-associated state (*ITGAX+*, *CST7+*, *TREM2+*, *SPP1+*) that persists into the chronic phase [6]. This finding has important implications for the therapeutic window of anti-inflammatory interventions.

Simultaneously, post-SCI ECM remodeling shifts the lesion toward an inherently inhibitory composition through up-regulation of CSPG family members, including neurocan, brevican, versican, aggrecan, and NG2, in reactive astrocytes and OPCs. These molecules show distinct temporal expression profiles: neurocan and versican peak at 2 weeks, whereas brevican remains elevated beyond 2 months [35]. CSPGs exert inhibitory effects by binding through their chondroitin sulfate chains to PTPσ and LAR receptor tyrosine phosphatases on axonal growth cones, activating RhoA/ROCK-mediated actin cytoskeletal contraction and growth-cone collapse. CSPGs also inhibit neural progenitor cell (NPC) neuronal differentiation through Wnt and FGF signaling, supporting CSPG receptor modulation as a therapeutic target [36,37].

Neurovascular pathology is critical in SCI but remains under-modeled in traditional experimental systems [38]. SCI triggers a complex vascular response that includes hemorrhage from disrupted microvasculature, vasospasm of surviving vessels, progressive microvessel loss through pericyte apoptosis and basement membrane degradation, and paradoxically limited post-injury angiogenesis despite increased VEGF expression [24,38,39]. In addition, inhibitory signals from the reactive glial scar and resultant spinal cord ischemia, together with excitotoxic and inflammatory secondary injury processes, contribute substantially to progressive tissue damage during the subacute phase [24,40]. In related BSCB-on-a-chip systems, this microvascular pathology can be modeled using BSCB compartments that include pericytes and astrocytes in addition to endothelial cells, allowing for the investigation of pericyte–endothelial cell crosstalk, MMP-9-mediated basement membrane remodeling, astrocytic endfoot retraction from vessels, and loss of paracellular selectivity in the disrupted BSCB [39,41]. Preconditioning with subthreshold inflammatory or ischemic stimuli before primary injury has been reported to protect against secondary injury in SCI models, but it has not been examined in human neural tissue because experimental pre-injury stimuli cannot be administered to humans. Currently, a prospective application of SCO-OoaC platforms may enable systematic study of neuroprotective preconditioning mechanisms in human spinal cord cellular models by allowing for precisely defined subinjurious stimuli, such as transient hypoxia, subtoxic glutamate pulses, low-level lipopolysaccharide exposure, or mechanical prestrain, to be administered before a defined injury protocol [26].

From a therapeutic-design perspective, the molecular complexity of the chronic SCI microenvironment suggests that single-target pharmacological approaches are unlikely to produce meaningful functional restoration, consistent with the historical experience of SCI clinical trials [42]. The post-injury niche promotes axonal growth-cone collapse through CSPG-PTPσ/LAR signaling, inhibits NPC neurogenesis through TGF-β- and BMP4-induced astrogliogenic bias, sustains neuronal apoptosis through TNF-α- and glutamate-induced excitotoxicity, and maintains an inhibitory ECM and M1-like microglial phenotype [37]. Therapeutic strategies may therefore need to target multiple pathological processes, either concurrently or in a defined temporal sequence. This creates a multidrug pharmacological challenge that requires experimental platforms capable of simultaneously tracking several biological readouts during combination-treatment regimens. SCO-OoaC platforms, with their multiparameter biosensing capacity related to the OoaC system and human-relevant cellular composition, may be well-suited to this drug-combination discovery application [2,43].

SCO-OoaC technology has the potential to reshape the conceptual framework of cellular-level preclinical SCI research. In this envisioned paradigm, the central question would no longer be only whether a therapy works in a rodent model, but whether it can restore human neural circuit function under human-specific molecular injury conditions [30]. This shift has important implications for the prioritization, characterization, and evaluation of drug candidates [44].

At the same time, SCO-OoaC technology and animal models should not be treated as competing approaches but as complementary tools in SCI research. In vivo SCI models remain necessary because they can recreate complex systemic responses to SCI, including hemodynamic responses, autonomic nervous system responses, peripheral immune-cell infiltration, musculoskeletal unloading effects, and behavioral outcomes, which are not yet fully reproduced by in vitro systems [45]. These hybrid technologies can therefore be described as advanced, human-relevant preclinical platforms that may help optimize and streamline drug development [46,47]. When used early in the development pipeline, SCO-OoaC platforms can test molecular targets in human tissue settings, screen compound libraries to identify potential therapeutics, define dose–response relationships in human cell types, and predict off-target effects unique to human biology. These methods may reduce reliance on resource-intensive and ethically sensitive in vivo models while aligning with the FDA’s 2025 regulatory direction and the 3Rs principles in animal research [48]. Finally, complementary integration of SCO-OoaC platforms into the preclinical research pipeline before animal experimentation may improve the translational relevance and predictive value of SCI therapeutic development while preserving the essential role of in vivo studies in validating safety, pharmacokinetic profiles, and functional outcomes [49].

## 3. Spinal Cord Organoids (SCOs): Structure and Fate Specification

### 3.1. Stem Cell Sources: Differentiation Protocols and SCO Generation

SCO generation uses two major stem-cell sources, each with distinct biological characteristics and translational implications [50]. First, human embryonic stem cells (hESCs), which are derived from the inner cell mass of preimplantation blastocysts, have strong pluripotency, reliable high-efficiency neural differentiation, and well-characterized quality-control standards; however, their use is limited by ethical concerns related to embryo use and by allogeneic immunological incompatibility, which may require human leukocyte antigen matching or lifelong immunosuppression in clinical applications [50]. Second, human induced pluripotent stem cells (hiPSCs), which are generated by reprogramming adult somatic cells with Yamanaka factors (OCT4, SOX2, KLF4, and c-MYC), have largely replaced embryonic stem cells as a source for SCO generation because they provide patient-specific genetic backgrounds, avoid embryo destruction, and support the clinical possibility of autologous or human leukocyte antigen-matched allogeneic cell products [51]. Sugai et al. (2021) previously reported the first human clinical trial of hiPSC-derived neural stem/progenitor cell (NS/PC) transplantation for subacute complete cervical SCI, outlining a clinical translation pathway for hiPSC technology and supporting the preclinical-to-clinical rationale for hiPSC-derived SCO applications [52]. Technically, good manufacturing practice (GMP)-compliant hiPSC generation using non-integrating episomal or mRNA-based reprogramming, xeno-free defined culture conditions, and full genomic-stability characterization is feasible, although manufacturing costs and scalability remain active engineering challenges for clinical-scale production [53].

### 3.2. Patterning Strategies: Neurogenesis of Rostro-Caudal/Dorso-Ventral Specification

SCO patterning strategies begin with neural induction through dual SMAD inhibition, achieved by blocking bone morphogenetic protein (BMP) signaling with LDN193189 or Noggin and inhibiting transforming growth factor-beta (TGF-β)/Activin pathways with SB431542. This approach enables efficient conversion of pluripotent stem cells into PAX6^+^/NESTIN+ neuroepithelial progenitor cells within 7–10 days [54]. However, dual SMAD inhibition alone biases differentiation toward an anterior, or forebrain, neuronal identity; therefore, active caudalization is required to generate spinal cord-specific phenotypes. Retinoic acid (RA; 0.1–1 μM), a key in vivo caudalizing morphogen secreted by the paraxial mesoderm, plays a central role in establishing posterior neural identity. When combined with CHIR99021, a glycogen synthase kinase-3beta (GSK-3β) inhibitor that activates the Wnt signaling pathway, RA promotes the specification of cervicothoracic spinal cord identity, as characterized by HOX gene expression [55]. The concentration, timing, and duration of RA and Wnt pathway activation critically regulate rostrocaudal patterning, enabling controlled generation of region-specific SCOs corresponding to cervical, thoracic, lumbar, and sacral spinal cord segments. These advances provide a robust platform for modeling level-specific SCI pathophysiology and may support the evaluation of region-targeted regenerative therapies [43,56].

Dorsoventral patterning of SCOs is governed by opposing morphogenetic gradients, primarily SHH signaling derived from the floor plate and antagonistic dorsal cues such as BMP signaling [43]. In vitro, ventralization is typically achieved by activating the SHH pathway with Smoothened agonists, including SAG (100–500 nM) or purmorphamine, which drive specification of ventral progenitor domains (p0-p3). Early work showed that coordinated dorsoventral patterning within individual organoids could be achieved by establishing simultaneous opposing SHH and BMP4 gradients. Xue et al. (2023) used composite porous chitosan microsphere–Matrigel@SAG scaffolds to recreate this spatial organization and enable robust dorsoventral domain formation [57]. Subsequent advances using microfluidic platforms further refined gradient control, allowing for precise spatial delivery of morphogens and revealing previously unrecognized crosstalk between RA and BMP4 signaling in dorsoventral boundary positioning. More recently, Xue et al. (2024) reported a microfluidic human stem cell-derived neural tube model that reveals pre-patterned axial identities of neural crest progenitors alongside comprehensive spinal progenitor specification [58]. This approach advances efforts to recreate human neural tube development in vitro and supports the use of SCO systems as models of developmental and injury-related processes.

### 3.3. Inducing Cellular and Functional Maturation of SCO

Recent SCO differentiation protocols generate a transcriptomically validated cellular repertoire that approximates the in vivo composition of the spinal cord along both dorsoventral and rostrocaudal axes [59]. The resulting populations include ventral motor neurons (ISL1+, HB9+, ChAT+, SMI32+), V0-V3 interneuron subtypes (EVX1/2+, EN1+, FOXN4+, SIM1+), dorsal sensory interneurons (PAX3+, Brn3a+, LBX1+, TLX3+), astrocytes (GFAP+, S100β+, AQP4+, GLAST+), oligodendrocyte precursor cells (OLIG2+, PDGFRα+, SOX10+), and radial glia (SOX2+, BLBP+) [60]. Single-cell RNA sequencing analyses have also shown that, with extended culture duration, these populations follow differentiation trajectories that converge toward primary human spinal cord transcriptomic reference datasets while preserving dorsoventral patterning fidelity, as defined by in vivo spatial gene-expression atlases [61]. Importantly, hiPSC-derived SCO neurons retain human-specific molecular features, including extended 3′ untranslated regions, distinct RNA-binding protein regulatory interactions, and species-specific differences in axonal transport machinery and growth factor receptor expression. These characteristics are largely absent from rodent primary neurons commonly used as experimental surrogates and may help determine human motor neuron susceptibility to SCI-related stressors and responsiveness to candidate therapeutics [62]. In addition, isogenic SCO pairs derived from patients harboring defined genetic variants, such as *STAT3*, *PTEN*, or the Nogo receptor, enable direct investigation of how human genetic variation affects post-injury axonal inhibition and glial reactivity. This capability supports precision-medicine applications that are not readily achievable in conventional rodent models [63].

Building on these advances, SCO maturation proceeds through a temporally coordinated sequence that recreates key stages of embryonic spinal cord development. Spontaneous electrophysiological activity typically emerges after 4–6 weeks in culture, as detected by microelectrode array recordings, and is followed by functional synaptic connectivity, evidenced by glutamatergic and GABAergic transmission in the form of spontaneous excitatory and inhibitory postsynaptic currents (sEPSCs and sIPSCs) [63]. Continued maturation is marked by the KCC2-dependent developmental shift in GABAergic signaling from depolarizing to hyperpolarizing responses, along with synchronized oscillatory bursting patterns characteristic of central pattern generator (CPG)-like network activity [60]. Building on this intrinsic maturation, assembloid strategies based on fusion of SCOs with region-specific organoids, such as cortical, peripheral sensory ganglion, and skeletal muscle tissues, have enabled reconstruction of higher-order, multilevel neural circuits, including corticospinal–motoneuronal–neuromuscular junction (NMJ) axes capable of optogenetically driven muscle contraction [45]. Importantly, such microfluidic platform might have enhanced physiological relevance by allowing for compartmentalized control of distinct neural regions and real-time functional interrogation. An important future direction, Luo et al. (2025) developed an ORDER-based strategy to generate patterned human neural tube organoids with well-defined dorsal–ventral patterning and electrophysiological activity, which might be enable the investigation of early human spinal cord development and the mechanisms of neurodevelopmental disease in a reliable model for the development of OoaC for the spinal cord [64]. Together, these developments establish SCO-based assembloid systems as an increasingly useful framework for studying human spinal cord circuit formation, function, and injury responses in a controlled, translationally relevant context [62].

### 3.4. Functional Validation of hSCOs

Beyond the morphological and transcriptomic validation, functional validation of hSCOs also shows that organoid-derived neurons form functional synaptic networks and reproduce important electrophysiological characteristics of the developing human spinal cord. This is validated by complementary structural and functional assays. Immunostaining of presynaptic and postsynaptic proteins (e.g., Synapsin-1 and Bassoon, PSD-95, VGLUT1/2, VGAT, and Gephyrin) shows the development of excitatory and inhibitory synapses, and whole-cell patch-clamp electrophysiology proves the existence of spontaneous excitatory and inhibitory postsynaptic currents (sEPSCs and sIPSCs), repetitive firing of action potentials, and membrane properties typical of spinal motor neurons and interneurons [60]. Further, neuromuscular junctions in both cortico-spinal and neuromuscular assembloid models suggest that hSCO-derived motor neurons can form physiologically relevant motor circuitry at the peripheral neuromuscular junction [45,62]. Together, these results suggest that hSCOs mature their synapses gradually and form functional neuronal networks to model the physiology and disease of the spinal cord [45,60,62].

Furthermore, methods of assessing network maturation include multielectrode arrays (MEAs) that allow for the non-invasive recording of spontaneous and evoked electrical activity over extended culture periods [65]. While complementary calcium imaging is used to visualize dynamics of the neuronal network ex vivo, optogenetic stimulation is used for cell-type-specific interrogation of circuit connectivity and functional integration. These methods provide a complete analysis of neuronal activity, synaptic transmission and network synchronization [66]. In the context of OoaC applications, a powerful way to optimize organoid selection for organoid functionality, before disease modeling and therapeutic screening, is to couple electrophysiological recordings with a non-destructive quality-control measure, such as cytokine profiling and live morphological imaging. While current hSCOs capture many key aspects of human spinal cord development, several aspects of long-term maturation, glial support, vascularization, and circuit complexity might still be improved to more faithfully recreate adult spinal cord physiology [66].

## 4. Organoids-on-a-Chip (OoaC): Mimicking the Spinal Cord Environment

An advanced in vitro platform known as organoid-on-a-chip has emerged by combining the complexity of 3D organoids with the microenvironmental control of microfluidics. OoaC platforms can recreate native tissue physiology more effectively than conventional 2D culture systems and may complement animal models. Earlier systems, including organ-on-a-chip platforms and assembloids, addressed some aspects of tissue organization but retained important limitations [67]. OoaC platforms are being developed to more faithfully reproduce the complex microenvironment of the human spinal cord.

Biomimetic engineering of the spinal cord microenvironment has become a central challenge at the interface of neuroscience, materials science, and microfluidic bioengineering. Traditional in vitro culture models cannot reproduce the dynamic biochemical gradients, mechanical stimuli, compartmentalized spatial architecture, and hemodynamic forces that collectively constitute the in vivo spinal cord niche. Many of these limitations can be addressed by integrating SCOs into OoaC microfluidic platforms, which provide sustained perfusion, spatially segregated culture chambers, defined ECM substrates, and real-time functional analysis within a single controlled experimental system. Recent advances in SCO-OoaC platforms have therefore produced some of the most physiologically relevant in vitro models for human spinal cord tissue engineering, and their continued development into integrated multicompartment neurovascular systems is shaping contemporary SCI research methods [16,68]. The following sections provide an overview of SCO-OoaC platform design and applications.

### 4.1. Microfluidic Chip Design and SCO Integration: Compartmentalization and Design

Spatial compartmentalization is a core engineering principle of OoaC platforms used to model the spinal cord. Single microfabricated devices can contain two or more distinct biological compartments in which the cellular composition, biochemical milieu, and mechanical stimulation of each compartment are independently manipulated while controlled and reproducible intercompartmental communication is maintained [69]. Operationally, compartmentalized neural devices use discrete culture chambers, typically representing soma, axon, glial, and vascular compartments, that are physically linked by arrays of size-restrictive microchannels. These channels are typically 5–10 μm wide and 3–5 μm high, allowing for selective axonal entry into distal compartments while excluding neuronal and glial cell bodies [68,70]. This geometry recreates the spatial segregation between neuronal somata in spinal gray matter and myelinated axonal projections in white-matter tracts. This feature is particularly relevant to SCI because axonal damage can be physically distinct from cell-body injury and mediated by different molecular pathways that may be targeted independently.

From a fluid-control perspective, microfluidic perfusion through embedded channels provides each compartment with oxygen, nutrients, and dissolved signaling factors at controlled rates and concentrations while removing metabolic waste products [71]. This fluid-flow microenvironment reproduces aspects of spinal cord interstitial-fluid circulation, prevents accumulation of inhibitory metabolites that limit the viability of static organoid cultures, and allows controlled fluid shear forces to be applied to vascular compartments that regulate endothelial barrier activity. Flow rates are generally regulated using off-chip syringe pumps or on-chip pneumatically actuated micropumps, and shear stress can be adjusted across a range of 0.001–23 dyn/cm^2^ by modifying channel geometry and operating conditions [44,71].

**Figure 2:**
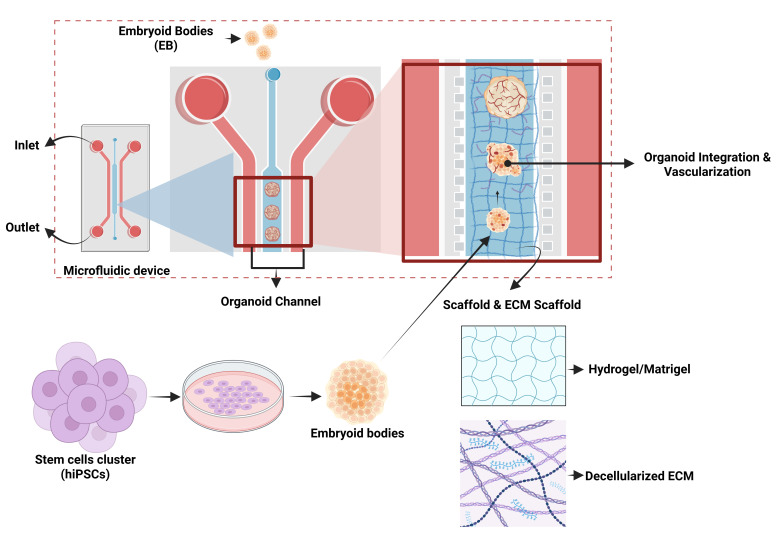
**Schematic illustration of biomimetic ECM hydrogels and an SCO-OoaC platform. **This figure illustrates the design and implementation of a microfluidic SCO-OoaC system for simulating the spinal cord microenvironment. To generate SCOs, stem cells are differentiated and cultured in the central chamber of a microfluidic device filled with ECM-based hydrogels. The platform enables controlled perfusion, nutrient exchange, and spatial organization, supporting organoid maturation and cell–matrix interactions under physiologically relevant conditions. Examples of hydrogel architectures are shown to illustrate how native spinal cord ECM can be modeled to provide structural and biochemical support for neural growth, differentiation, and disease modeling. Created in BioRender. Roh, E. (2026) https://BioRender.com/9uaxbwt (Agreement number: BJ29TPAI5P).

Three main strategies have been developed to incorporate SCOs into OoaC devices, each offering distinct experimental advantages. The first is direct seeding, in which intact organoids produced by standard suspension culture protocols are transferred into preformed chip chambers. This approach preserves 3D cytoarchitecture and self-organized cellular heterogeneity but may compromise reproducible positioning and contact with device surfaces. Ao et al. (2022) directly loaded human pluripotent stem cell-derived sensory-spinal cord organoids into a 3D-printed air–liquid interface organoid-holder chip, preserving capsaicin- and mustard oil-evoked nociceptive circuit architecture and enabling plug-and-play microelectrode array electrophysiological recording of neuronal spiking [72]. The second strategy is dissociation–reconstitution, in which organoids are enzymatically dissociated into single-cell suspensions, combined with hydrogel bioinks, and reconstituted in chip compartments [68]. Xue et al. (2023) showed that dissociating dorsoventral-patterned SCOs and reconstituting the single-cell suspension in a composite porous chitosan microsphere–Matrigel@SAG scaffold loaded into a chip chamber produced spatially controlled reorganization of NKX6.1+ ventral and PAX3^+^ dorsal progenitor domains, which was not achievable with intact organoid seeding alone [57]. Finally, pluripotent stem cells can be cultured using an in situ differentiation approach, in which cells are seeded directly into chip chambers and differentiated under microfluidic flow conditions [73]. This approach permits real-time monitoring of morphogenetic processes and continuous exposure to defined flow-mediated developmental cues, but it requires lengthy on-chip culture protocols and technically demanding quality control (Figure 2). The choice of integration strategy depends on the experimental objective, device geometry, and developmental stage at which OoaC interaction is intended to begin [68].

### 4.2. ECM Scaffolds: Matrigel and Beyond

The extracellular matrix scaffold has a fundamental influence on organoid self-organization, cellular differentiation pathways, mechanosensing signals, and long-term structural stability in SCO culture. Matrigel, a growth factor-reduced basement membrane extract from Engelbreth-Holm-Swarm murine sarcoma, remains the scaffold of choice in many current organoid studies [74]. However, it is poorly suited to translational SCO-OoaC applications for several reasons: its xenogeneic murine origin and intrinsic immunogenic potential, chemically undefined and lot-dependent heterogeneous composition, failure to recreate the tissue-specific proteoglycan and growth-factor composition of the human spinal cord ECM, and lack of mechanical tunability, which limits systematic investigation [74,75]. These limitations have driven the development of defined biomaterial substitutes, including synthetic hydrogels, natural biopolymers, and decellularized extracellular matrix-derived materials. Each option offers distinct advantages in reproducibility, biological authenticity, and engineering controllability.

#### 4.2.1. Functional Hydrogels: Hyaluronic Acid and GelMA Systems

Hyaluronic acid (HA) is a natural polymer hydrogel with distinctive biological relevance in spinal cord tissue engineering. HA is the most prevalent non-sulfated glycosaminoglycan in the central nervous system extracellular environment, a major component of perineuronal nets that regulate synaptic plasticity and neuronal excitability, and a mechanically compliant material with an elastic modulus (~0.1–1 kPa) that approximates native spinal cord parenchymal rigidity [76]. As a neural progenitor culture scaffold, HA facilitates neural stem cell (NSC) adhesion, process extension, and neuronal differentiation while suppressing astrocytic fate bias, which is directly relevant to the astrogliogenic pressure exerted by the post-SCI glial scar. Through thiolation, methacrylation, or aldehyde modification, HA can also be modified to allow for controlled crosslinking, incorporation of bioactive peptides (IKVAV, YIGSR, and RGD), and spatiotemporally controlled degradation kinetics comparable to tissue-regeneration timescales [77]. In a representative study, Pereira et al. (2023) reported that methacrylated HA bioinks containing GelMA as a structural reinforcer formed neural progenitor cell constructs with enhanced long-term neuronal differentiation compared with GelMA alone, and PAX6 expression increased over time in 3D-printed constructs, supporting the utility of HA-GelMA composites as defined scaffold systems for SCO-OoaC applications [77].

In SCI repair, GelMA hydrogels are among the most extensively used and flexible defined scaffold materials in organoid bioengineering. They provide ultraviolet-crosslinkable and continuously tunable stiffness (1–30 kPa) through adjustments in polymer concentration and ultraviolet exposure duration, show high cytocompatibility, and contain RGD integrin-binding and MMP-cleavable sequences that facilitate cell adhesion and matrix remodeling [78]. GelMA stiffness tuning is physiologically relevant to SCI applications because the transition from native spinal cord parenchyma (~0.5 kPa) to consolidated glial scar (~315 kPa) activates YAP/TAZ mechanotransduction pathways that bias NSC fate toward astrogliogenesis [79]. Recently, Li et al. (2025) reported that hiPSC-generated hSCOs, produced through stage-specific patterning involving dual SMAD inhibition and motor neuron specification via RA and SAG, were encapsulated in GelMA hydrogel before implantation into a rat SCI model [80]. GelMA-wrapped hSCOs improved neural regeneration and restored motor activity in rat SCI, providing in vivo evidence that GelMA-SCO composite constructs have biological activity relevant to functional repair [80].

#### 4.2.2. Decellularized Extracellular Matrix (dECM) Scaffold: Native ECM Scaffolds

Decellularized extracellular matrix (dECM) hydrogels are produced by removing cellular and nuclear components from native tissue while retaining the three-dimensional protein scaffold, proteoglycan network, incorporated growth factors, and tissue-specific signaling molecules [81]. These hydrogels are among the most biochemically realistic scaffold materials currently available for SCO-OoaC experiments because they capture the compositional complexity of spinal cord ECM more fully than synthetic or individual-protein hydrogels. Spinal cord-specific dECM hydrogels are generally prepared by enzyme-mediated decellularization of porcine or human spinal cord tissue using enzymatic-detergent protocols, followed by pepsin solubilization and thermal gelation at 37 °C. This process preserves native ECM components such as collagen IV, laminin isoforms, fibronectin, heparan sulfate proteoglycans, and CSPG family members, including neurocan, brevican, and aggrecan, which are substantially increased in reactive astrocytes after SCI [82,83].

In vivo studies have supported the neuroregenerative potential of spinal cord dECM. Tukmachev et al. (2016) reported that injection of porcine spinal cord-derived ECM hydrogel into acute hemisection SCI cavities promoted neovascularization and axonal growth and reduced subsequent cystic cavity formation at multiple post-injection time points [82]. Several similar studies have shown that spinal cord dECM hydrogels loaded with NSCs can enhance functional recovery and remyelination in rodent SCI models by improving the survival and differentiation of transplanted neurons. For example, another study by Liu et al. (2024) also confirmed motor functional recovery [84]. Similarly, Sun et al. prepared a dECM hydrogel (DNSCM) that promoted neural progenitor proliferation, SCO maturation, axonal regeneration and neuronal differentiation. Finally, transplantation of hydrogel with neural progenitor cells has significantly improved functional recovery in SCI models [85].

ECM-based composite scaffolds with GelMA have also been fabricated by electrospinning to produce aligned nanofibrous structures that replicate the structural anisotropy of native ECM. Compared with GelMA alone, these scaffolds have been shown to promote NSC neuronal differentiation and reduce proinflammatory M1 macrophage polarization [86]. More recently, Yan et al. (2025) suggested a conductive dECM/GelMA composite hydrogel that, in combination with electrical stimulation, promoted axonal regeneration, improved electrophysiological function, and enhanced locomotor recovery following spinal cord injury [87]. Although dECM hydrogels are biological, they have several intrinsic limitations that limit their ability to be reliably used in SCO-OoaC platforms. Partial loss of key bioactive proteins such as growth factors (FGF-2, EGF, BDNF), glycosaminoglycan chains (heparan sulphate, HA) and labile extracellular matrix proteins inevitably occur during the decellularization process. This loss is dependent on the parameters of the decellularization process and cannot be fully standardized amongst different laboratories [88,89]. Variability related to donor-to-donor differences in tissue source arising from differences in donor age, sex, disease status, or post-mortem interval for human-derived dECM or inter-animal biological variation introduces batch-dependent compositional heterogeneity in proteoglycan content, collagen crosslinking density and residual growth factor levels that compromises experimental reproducibility, as would Matrigel [83]. Additionally, the limited availability of human spinal cord tissue, limited by ethical concerns, regulatory requirements and low post-mortem tissue yield, significantly limits the scalability of human-specific spinal cord dECM production, making xenogeneic porcine dECM the practical default although it has immunogenic potential and differs in species composition to human spinal cord ECM [90]. All these constraints highlight the need for complementary defined scaffold strategies that retain the biological instructive benefits of dECM and offer compositional uniformity and scalability for standardized SCO-OoaC applications.

### 4.3. OoaC Systems for Modeling the Neurovascular Unit and Blood–Spinal Cord Barrier

#### 4.3.1. The BSCB as a Therapeutic Target and Engineering Challenge

The BSCB, which comprises non-fenestrated endothelial cells sealed by tight-junction complexes (claudin-5, occludin, ZO-1, and ZO-2), periendothelial pericytes surrounding capillaries, and astrocytic endfeet covering the abluminal surface, is a key target of secondary SCI pathology [26]. Acute BSCB injury occurs within minutes after primary mechanical trauma, when MMP-2/9-mediated proteolysis of tight-junction proteins facilitates the extravasation of plasma proteins, erythrocytes, and circulating immune cells into the spinal cord parenchyma. Pharmacological restoration of BSCB integrity is therefore a well-supported but difficult therapeutic goal, and systematic testing requires experimental systems that realistically reproduce the multicellular BSCB structure under physiologically relevant hemodynamic conditions. These requirements remain incompletely met by current OoaC models of the neurovascular apparatus [41].

#### 4.3.2. Functional Validation of BSCB-on-a-Chip

Recent BSCB-on-a-chip systems have improved barrier-function fidelity by systematically optimizing cell sources, culture configurations, and hemodynamic conditions [91]. Systems using induced pluripotent stem cell-derived endothelial cells, pericytes, and astrocytes under controlled shear stress (10–20 dyn/cm^2^) [92] achieve transendothelial electrical resistance (TEER) values of 500–2000 Ω·cm^2^ [93] and paracellular dextran (10 kDa) permeability coefficients of 10^−7^–10^−8^ cm/s, approaching values measured in primary in vivo spinal cord microvessels and substantially exceeding those of static Transwell cultures [92,94,95]. The mechanistic role of shear stress in establishing a barrier phenotype has been demonstrated across several platforms: shear stress induces endothelial-cell elongation and flow-aligned morphology, activates Krüppel-like factor 2 (KLF2)-mediated mechanosensitive transcription, upregulates tight-junction proteins [96,97], and alters the inflammatory gene-expression profile of statically cultured endothelial cells. This is a tri-cellular tight junction regulatory program that is temporally coordinated by dynamic flow conditions and only these conditions can fully initiate and sustain the program [98]. Within hours of onset of shear, KLF2 translocates to the nucleus and leads to up-regulation of claudin-5 and ZO-1; shear stress leads to significant up-regulation of claudin-5 and ZO-1. This shear-initiated program is further supported by pericyte-secreted angio-poietin-1 (Ang-1), which stabilizes occludin-ZO-1 interactions via Tie-2/PTPN-2 signaling, and by astrocyte endfoot-secreted TGF-β, which further amplifies claudin-5 expression [98,99].

Building on this work, a previous study introduced TNF-α-activated or SCI-conditioned microglia into the neural compartment of a BSCB-on-a-chip device and showed that TNF-α induced endothelial necroptosis and downregulated claudin-5 and ZO-1 in the neighboring vascular compartment, directly recapitulating a microglia-mediated mechanism of BSCB disruption established in in vivo SCI studies [91]. In addition, the molecular mechanism of this injury-induced tight junction disruption is revealed by the fact that treating BSCB-on-chip devices with inflammatory stimuli that are relevant to SCI, such as TNF-α, releases claudin-5 and ZO-1 into the extracellular environment in a manner that is reversible by the broad-spectrum MMP inhibitor GM6001 and quantifiable by measuring the decrease in TEER and the loss of immunofluorescent junction strand continuity [100], establishing a chip-specific pharmacological screening paradigm for BSCB-protective therapeutics targeting the degradation cascade of claudin-5 and ZO-1 [101]. Importantly, the comparison of flow versus static reveals that the disruption of tight junctions takes place over 24–48 h in static co-culture, whereas it takes place within 4–6 h in flow-conditioned OoaC, which is concordant with the acute hemodynamic phase of in vivo SCI BSCB disruption, showing that dynamic OoaC configurations are required to reproduce the kinetics of the injury necessary for acute-phase pharmacological evaluation [102,103].

Interior vascularization of organoids is also needed to provide metabolic support to organoids larger than 400–600 μm in diameter, which otherwise develop necrotic cores because of diffusion-limited hypoxia. Previously, Cakir et al., introduced a novel method for creating vascularized human brain organoids using human embryonic stem cells (hESCs) that express a constitutively active form of the transcription factor ETV2 and a wild-type hESC aggregate. Upon ETV2 induction, these cells differentiated into endothelial cells that self-organized into vascular-like networks with lumen formation, expressed brain endothelial markers, and exhibited dextran perfusion. Non-vascularized and vascularized organoids exhibited certain differences with their reduced hypoxia, improved neuronal maturation, and improved cell viability [104]. Consistent with this approach (Table 1), SCO-OoaC systems have been engineered by embedding SCOs within fibrin hydrogels containing induced pluripotent stem cell-derived endothelial cells, pericytes, and proangiogenic growth factor cocktails, including vascular endothelial growth factor A (VEGF-A), basic fibroblast growth factor (bFGF), and sphingosine kinase 1 (SphK1) activators. These components promote spontaneous self-assembly of perfusable capillary-like vascular networks capable of anastomosing with microfluidic inlets [20]. Notably, a recent study showed that two-photon polymerization 3D printing can fabricate geometrically accurate perfusable microvascular scaffolds with diameters smaller than the tissue diffusion limit (~100 μm), providing a deterministic template for endothelial-cell seeding and vascular-network formation within organoid constructs without relying on stochastic vasculogenic self-assembly [70].

## 5. Advanced Applications of SCO-OoaC in SCI

A recent study reported that combining SCOs with an OoaC system provides a hybrid experimental model that extends beyond conventional tissue culture methods. SCO-OoaC models can serve as dynamic platforms for studying cell–biomaterial interactions in regenerative microenvironments, testing extracellular vesicles as cell-free therapies, mapping neural-network regeneration over time and space, and examining gene–environment interactions using clustered regularly interspaced short palindromic repeats (CRISPR)-based techniques. These applications extend in vitro neural tissue engineering toward systems with greater physiological function and therapeutic relevance [43,44].

Additionally, the injury paradigms employed in SCI models illustrate distinct aspects and temporal phases of spinal cord injury and should not be regarded as interchangeable representations of traumatic SCI [125]. These paradigms can be broadly classified into four functional categories: (i) acute excitotoxic and metabolic injury paradigms [29], including localized glutamate exposure, oxygen–glucose deprivation (OGD), hypoxia, and hydrogen peroxide-induced oxidative stress, which mimic the hyperacute and acute secondary injury cascade (0–48 h post-injury); (ii) neuroinflammatory injury paradigms, which recreate the subacute inflammatory phase (2 days–2 weeks post-injury); (iii) chronic inhibitory microenvironment paradigms, including CSPG-rich extracellular matrix conditions and glial scar-mimetic scaffolds [126], which reproduce the inhibitory environment that limits axonal regeneration during the chronic phase (weeks to months post-injury); and (iv) functional circuit dysfunction models, including nociceptive circuit activation and BSCB disruption assays, which model specific pathological consequences of SCI rather than the injury cascade itself [117]. These paradigms are discussed in the following subsections. Although each reproduces a distinct component of SCI pathophysiology, no currently available SCO-OoaC platform integrates all these features within a single system, and achieving such comprehensive multi-component modeling remains an important objective for future platform development.

### 5.1. Cell–Biomaterial Interactions and Axonal Regeneration

SCO-OoaC systems enable systematic investigation of how biomaterial characteristics, including stiffness, composition, surface morphology, and chemistry, influence NSC fate in the inhibitory post-SCI environment. Activation of the PI3K/Akt signaling pathway by nerve growth factor NT-3-loaded silk fibroin hydrogels and magnetomechanical stimulation has been identified as a key mechanism promoting neuronal rather than astroglial NSC differentiation [27,127]. In a recent translational study, Han et al. (2025) used 3D-printed spinal organoid scaffolds incorporating precisely sized microscale channels seeded with iPSC-derived region-specific spinal neural progenitor cells (sNPCs) and demonstrated directional axonal extension within the channels, promoted neuronal maturation and the circuit network, and improved locomotor outcomes after transplantation into transected rat spinal cords [128]. In OoC-based injury models, biomimetic CSPG hydrogel barriers can represent the inhibitory ECM component of the glial scar, while sequential application of enzymatic and ligand-targeting treatments, such as ChABC, ISP, and ILP, can support preclinical testing of candidate scar inhibitors [129].

### 5.2. Extracellular Vesicle Therapeutics

Extracellular vesicles (EVs) are among the most promising cell-free therapies for SCI. EVs can penetrate the BSCB, deliver multiple bioactive molecules, including microRNAs, long noncoding RNAs, proteins, and lipids, and regulate several pathological pathways with low immunogenicity [130,131]. According to the Minimal Information for Studies of Extracellular Vesicles 2023 standards from the International Society for Extracellular Vesicles, EVs are classified as exosomes (30–200 nm) or large EVs (>200 nm). In meta-analyses of preclinical SCI studies, mesenchymal stem cell (MSC)-derived EV administration has repeatedly been shown to improve locomotor outcomes, shift M1/M2 microglial polarization, prevent neuronal apoptosis, and promote axonal regeneration and BSCB repair [132]. Furthermore, such preclinical studies demonstrate that the therapeutic potential of the MSC-derived EVs at the level of the organism and focus on functional outcomes like locomotor recovery, lesion volume, and tissue repair, providing only a basic level of mechanistic resolution of human-specific cellular responses [133]. SCO-OoaC platforms in contrast represent a complementary, human-relevant microphysiological system allowing for investigations of EV-mediated effects in a microenvironment setting of well-defined conditions [16]. When combined with microfluidic perfusion, SCO-OoaC systems allow for real-time evaluation of neuronal survival, axonal regeneration, electrophysiological activity and the integrity of the BSCB after EV treatment. However, current SCO-OoaC platforms cannot imitate the pharmacokinetics, peripheral immune interactions, or behavioral recovery of in vivo research. Therefore, these platforms should be considered as translational tools to fill the gap between traditional in vitro experiments and animal models, aiming to provide mechanistic insight into the pharmacodynamics of EVs and optimize EV-based therapeutics for in vivo validation [16,43].

Mechanistically characterized EV microRNA cargoes include miR-216a-5p from hypoxic preconditioned MSC EVs, which stimulates M1/M2 transition through JAK2-STAT3 signaling; miR-340-5p from umbilical cord MSC EVs, which regulates M1/M2 microglial phenotype switching through JAK/STAT3 signaling; miR-692 from brown adipose tissue-derived EVs, which inhibits Spp1 expression in microglia, as shown by positron emission tomography/computed tomography analysis of brown adipose tissue activation after SCI; and miR-222-3p from endothelial progenitor cell EVs, which influences M2 polarization and angiogenesis through SOCS3/JAK2/STAT3 signaling. Engineered nanovesicles termed Treg–Exo–IKVAV, which are cell-derived exosomes covalently linked to the neurogenic laminin peptide IKVAV, provide an example of a multifunctional next-generation EV therapy that combines early-phase immunomodulation with axonal regenerative effects [134]. SCO-OoaC technology enables mechanistic investigation of EV pharmacodynamics through longitudinal tracking: cytokine biosensors can measure M1/M2 polarization changes, MEA can detect functional network recovery, and spatial transcriptomics can characterize transcriptional changes across organoid regions.

### 5.3. Spatiotemporal Mapping of Neural Network Regeneration

A key advantage of SCO-OoaC technology is the ability to monitor axonal growth and synaptogenesis longitudinally and spatially in human-derived neural tissue. Fluorescent axonal labeling combined with live-cell imaging in a microfluidic platform can reveal the speed, direction, and branching patterns of axonal growth after defined chemical or mechanical stimulation [78]. Together, SCOs and microfluidic compartmentalized circuits enable quantification of axonal growth rates, approximately 0.1–1 mm/day in central neurons, and identification of factors that influence directional migration [135].

#### 5.3.1. Electrophysiological Recovery: MEA and Calcium Imaging

**Figure 3:**
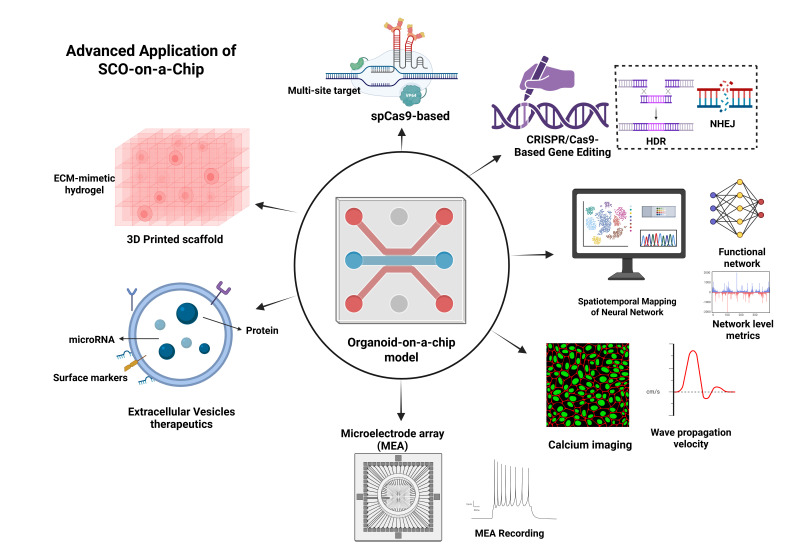
**SCO-OoaC platforms: advanced applications. **Schematic illustration of representative applications of SCO-OoaC systems in neural engineering and translational neuroscience. The platform integrates biomimetic 3D-printed ECM hydrogels, EV-based therapeutics, CRISPR/Cas9-mediated gene editing, and electrophysiological monitoring with MEAs. Functional assessment methods include calcium imaging, spatiotemporal neural-network mapping, wave-propagation analysis, and network-level activity measurement to assess neuronal connectivity and function. These technologies can support disease modeling, drug screening, regenerative medicine research, and precision therapeutic development. Created in BioRender. Roh, E. (2026) https://BioRender.com/4w982yt (Agreement number: GN29TPAS4I).

MEA recording and calcium imaging are two major approaches for functionally characterizing neuronal network activity in SCI-on-a-chip systems, allowing for quantification of electrophysiological recovery after artificial injury and therapeutic intervention. Additionally, MEA technologies range from conventional planar microelectrode arrays to recently developed suspended mesh MEAs by McDonald et al. and kirigami-based flexible electronics Yang et al., which enable long-term recording of spontaneous action potentials, local field potentials, and neuronal network activity in human neural organoids and assembloids. These technologies provide promising platforms that could be adapted for future electrophysiological assessment of spinal cord organoids [65,66]. Among on SCI-on-a-chip models, mechanical sectioning or local delivery of glutamate neurotoxin to organoids in a microfluidic system produces electrophysiological changes, such as decreased spontaneous firing, reduced synchrony, and lower spike rates recorded by MEA (Figure 3), which can then be monitored during treatment administration [16].

Calcium imaging with genetically encoded calcium indicators, such as GCaMP variants (GCaMP6s, GCaMP7f, and jGCaMP8), delivered under cell-type-specific promoters (all neurons: Synapsin; motor neurons: ChAT; V1 interneurons: EN1), offers single-cell resolution for detecting spontaneous and stimulus-induced neuronal activity in SCOs, complementing population-level MEA measurements [78]. In addition, optogenetic delivery of channelrhodopsin-2 (ChR2), halorhodopsin, or soma-targeted derivatives through viral vectors or CRISPR-mediated knock-in approaches allows for selective activation of motor neurons and/or interneurons and measurement of downstream circuit responses. This approach is well-suited for evaluating synapse formation and restoration of synchronized circuit activity in SCO-OoaC systems after cycles of injury and recovery [136].

In the context of long-term culture of SCO-OoaC, current electrophysiology monitoring approaches have their own limitations. While the planar MEAs offer label-free and non-invasive recordings at very high temporal resolution, the capacity to record is limited to the cells placed in the vicinity of the electrode surface, thus leading to under-sampling of the neuronal activity of the deeper parts of 3D organoids [137]. In a similar manner, calcium imaging can be used to observe the network dynamics and single-cell activity at high spatial resolution but requires repeated fluorescence excitation and suffers from the problem that prolonged imaging may lead to photobleaching, phototoxic effects, and changes in calcium homeostasis, making it difficult to study chronic cell injury and long-term neuronal maturation [138]. Despite the advantages of optogenetics for the control of neuronal activation and inhibition, it is not an easy tool to use, requiring genetic manipulation of targeted cells, and is restricted by low light penetration and optical scattering in dense tissue layers of large organoids. At present, however, a single modality does not offer a full functional characterization of long-term cultures of SCO-OoaC; thus, a combination of electrophysiology monitoring approaches is required [138].

Furthermore, recent developments in bioelectronic interfaces have centered on multimodal platforms that integrate complementary approaches like using MEAs, calcium imaging, optogenetic stimulation and microfluidic biosensors to measure both electrical activity and changes in the microenvironment and intracellular signaling. One of these advancements is kirigami-inspired flexible electronics for long-term organoid electrophysiology [66]. In contrast to traditional planar MEAs, ultrathin kirigami electrodes change from a flat surface to a 3D basket-like structure that wraps around suspended organoids, ensuring stable electrical contact without disrupting tissue morphology and self-organization [138]. Such a design allows for chronic electrophysiological recording for over 100–120 days, enhances the acquisition of signals from 3D neuronal networks and is compatible with both optogenetic stimulation and pharmacological perturbation. Thus, flexible bioelectronic interfaces provide a promising approach for future platforms of the SCO-OoaC by providing the means to longitudinally assess multimodal functional development, injury development, and therapeutic responses in a physiologically relevant 3D environment.

#### 5.3.2. CRISPR/Cas9-Based Gene Editing in SCO-OoaC Systems

The integration of CRISPR/Cas9 technology, its variants, and related precision gene-editing techniques with SCO-OoaC models represents an important methodological frontier in SCI research [139]. These approaches enable analysis of gene–environment interactions in human-specific 3D neural tissue under tightly controlled environmental conditions. The CRISPR-based toolkit has moved beyond the initial use of SpCas9 to generate double-strand breaks and now includes eSpCas9 and HiFi-Cas9 (Figure 3), which minimize nonspecific cleavage in neuronal cells [140]; Cas12a/b systems with improved protospacer adjacent motif specificity; Cas13 RNA-targeting systems that enable reversible and temporally controlled RNA editing without DNA editing; base editors that can precisely convert single nucleotides without double-strand breaks; and prime editors that enable template-free DNA modification [139,141].

For motor neuron diseases, CRISPR/Cas9 technology has been used to generate isogenic SCOs from patient-derived iPSCs carrying amyotrophic lateral sclerosis-related mutations in genes such as SOD1, FUS, TARDBP, and C9orf72. This method allows for direct comparison of mutant and gene-corrected organoids in the same microfluidic environment and supports the investigation of gene-specific pathogenesis. Moreover, gene correction in SCO-derived motor neurons rescues RNA foci, dipeptide-repeat protein accumulation, and the hyperexcitability phenotype detected through MEA recordings [142,143]. CRISPR activation and CRISPR interference allow for regulation of gene expression without altering the genome in SCOs and can help identify transcriptional networks, such as STAT3 in reactive astrocytes, NF-κB in microglia, and YAP/TAZ in mechanosensitive progenitors, that control cell phenotype.

## 6. Current Challenges, Limitations, and Engineering Bottlenecks

Despite advances in SCO-OoaC technology, substantial biological and engineering bottlenecks continue to limit physiological fidelity, reproducibility, scalability, and translational applicability. These constraints reflect the difficulty of recapitulating the structural, cellular, biochemical, and biomechanical complexity of the native spinal cord microenvironment in an in vitro platform. This section discusses limitations in vascular integration, multicellular organization, long-term maturation, immune competence, biomechanical simulation, and standardized fabrication methods [123,144].

### 6.1. Incomplete Physiological Complexity

#### 6.1.1. Immune-Integration Gap

The absence of authentic immune-cell populations, including resident microglia and peripheral immune cells that mediate secondary injury after SCI, is a major biological limitation of current SCO-OoaC platforms. While human-derived iPSC microglia suggested the expression of canonical homeostatic markers such as P2RY12, TMEM2, and CX3CR1, their transcriptomic signatures and functional responses do not completely overlap with adult primary human microglia [145,146]. Notably, in a single-cell study of SCI, Apoe, Lpl, Cst7, Trem2, and Spp1 are all expressed by microglial populations similar to amoeboid DAMs, but these models only partially replicate pathological states found in vivo [147]. Thus, there is a need for additional refinement of microglial differentiation protocols and for the use of multicellular and microenvironmental cues to better achieve homeo- and injury-associated phenotypes of adult microglia [148,149]. In addition, subacute SCI pathology, which depends on peripheral immune-cell infiltration through CCR2+ monocyte diapedesis, neutrophil transendothelial migration, and T-cell-mediated neuroinflammatory signaling, is not represented in most currently available SCO-OoaC platforms. This underrepresentation of the inflammatory microenvironment is arguably one of the most important biological constraints for SCI drug-development applications [135,150,151].

Furthermore, although recent developments have allowed for the use of iPSCs to generate microglia, existing platforms for generating SCOs and microfluidics have not been able to model the complex peripheral immune response observed during SCI [152]. After BSCB disruption, infiltrating neutrophils, monocyte-derived macrophages, T lymphocytes, B lymphocytes and dendritic cells interact with resident microglia and neural cells and mediate secondary injury, neuroinflammation, scar formation, and tissue repair [153]. Yet, the stable incorporation of these peripheral immune populations into SCO-OoaC systems is still technically challenging due to their unique culture requirements, limited long-term viability, problems with reproducing immune-cell recruitment and transmigration across the BSCB, as well as the lack of a fully perfusable neurovascular interface [133]. Thus, existing models of SCI in the immune system are not fully representative of the multicellular interactions that contribute to disease progression. The further development of controlled perfusion and recruitment of immune cells into BSCB modules will facilitate the modeling of the role of the immune system in the brain and enhance the predictive potential of SCO-OoaC platforms in screening for immunomodulatory therapies.

#### 6.1.2. Vascularization Deficiency

In organoids lacking perfusable vasculature, inner-core hypoxia can compromise viability, metabolic function, and biological authenticity during extended culture, particularly in organoids larger than 400–600 μm in diameter; these features are critical for maturation and chronic SCI modeling [154]. Relevantly, a study by Cakir et al. demonstrated that as human brain organoids increase in size during long-term culture, limited oxygen and nutrient diffusion results in hypoxia and central apoptosis, as evidenced by TUNEL staining. By engineering vascular-like networks within the organoids, the authors reduced hypoxia and improved cell viability, highlighting the importance of vascularization for maintaining large and structurally complex organoids [104]. Both vascularization strategies, ETV2 endothelialization and fibrin-embedded vasculogenesis, have established proof of concept, but their vascular networks remain stochastic, BSCB tight-junction expression within organoid interiors is inconsistent, and reliable anastomosis with external microfluidic perfusion circuits has not yet been achieved [154].

### 6.2. Standardization and Reproducibility: Organoid Heterogeneity

A major issue in current SCO-OoaC applications is the lack of standardized protocols and system validation. Variability in SCO size, composition, regional identity, and electrophysiological maturity across batches and laboratories remains a major practical barrier to translating SCO-OoaC technology into robust drug-screening and disease-modeling tools [144,155]. This variability arises from several sources, including stochastic morphogen gradients during initial self-organization, batch-to-batch variability in Matrigel, donor-to-donor variability in iPSCs, and incubator-to-incubator variability in organoid culture. Beyond donor-to-donor variability, iPSCs also have intrinsic biological and technical challenges that impact the reproducibility of the SCOs [91]. These include genomic instability during extended passaging, spontaneous differentiation due to incomplete maintenance of pluripotency, differences in the efficiency of neuronal differentiation from different neural stem cell lines, and epigenetic memory from the donor neural stem cell of origin, which can affect neural patterning, cellular composition, and functional maturation of SCOs [91]. To overcome these limitations, stringent quality control procedures such as genomic integrity testing, quantification of pluripotency and validation of organoid differentiation potential are essential [156]. These factors can produce organoids within a single batch that differ in developmental stage, dorsoventral neuron subtype ratios, or network electrophysiology, thereby undermining the statistical power of pooled functional measurements [144,155]. Potential remedies include replacing Matrigel with batch-independent chemically defined hydrogels, using microfluidic embryoid body formation to generate size-controlled precursor organoids, and automating differentiation protocols with real-time quality-control biosensing. These approaches are being incorporated into the CEN/CENELEC OoC Standardization Roadmap (2024) and the NIST-led working group on OoC standards, which together aim to establish minimum reporting requirements, interlaboratory microfluidic chip validation tests, and performance standards for regulatory submission of SCO-on-a-chip-derived pharmacology data [156,157].

### 6.3. Microfluidic Fabrication Scalability Challenges

The most common microfluidic substrate is polydimethylsiloxane (PDMS), which is optically transparent and can be rapidly prototyped using soft lithography. However, PDMS has well-known drawbacks that are particularly relevant to SCO-OoaC systems, including adsorption of small hydrophobic molecules, such as morphogens, neurotrophic factors, and drugs, at concentrations that can interfere with dose–response curves; high gas permeability that affects dissolved oxygen gradients; and manual fabrication processes that produce interchip geometric variation and compromise fluidic predictability [123]. Alternative microfluidic materials include thermoplastics, such as cyclic olefin copolymer, polystyrene, and polymethyl methacrylate, which have superior chemical inertness and can be rapidly produced by injection molding for high-throughput applications but require expensive tooling and lack rapid prototyping flexibility [158]. Scaling SCO-OoaC fabrication to tens or hundreds of parallel chips, as required for regulatory-grade drug testing, remains an unresolved engineering challenge. This issue has become more important as recent FDA initiatives have encouraged the incorporation of human-relevant new approach methodologies, including organoids and OoC systems, into drug development and safety evaluation [123].

### 6.4. Functional Assays: Need for High-Resolution Real-Time Monitoring

Current SCO-OoaC readouts rely heavily on invasive endpoint analyses, such as immunohistochemistry (IHC), scRNA-seq, and Western blotting, which cannot capture dynamic pathology or pharmacodynamics in individual organoids over the timescales relevant to SCI therapeutic studies. Simultaneous real-time measurement of inflammatory cytokines, neurotrophic factors, metabolites, and electrophysiological network activity within a single SCO-OoaC device remains a critical technical hurdle. Printed circuit board (PCB)-based electrochemical immunosensor arrays capable of simultaneous eight-analyte detection offer a near-term technical solution, with modular integration into OoaC effluent channels that does not interfere with optical monitoring or organoid health [159,160]. In addition, limited optical penetration of confocal microscopy in 3D organoid tissue requires tissue clearing protocols, such as iDISCO^+^ and CUBIC, or alternative imaging and recording strategies, such as light-sheet microscopy, two-photon excitation, and malleable injectable 3D microelectrode arrays, for multidimensional mapping of neural activity. The limited maturation achieved by current SCO protocols constrains modeling of chronic SCI pathology, including mature glial scar formation, late-stage Wallerian degeneration, and mature neuropathic pain synapses. Longer culture periods, the GENtoniK maturation cocktail [161], and forced maturation through targeted small-molecule cocktails that activate epigenetic, Ca^2+^-dependent, and synaptic maturation pathways are being pursued to overcome this limitation.

Although MEA electrophysiology, GCaMP calcium imaging, and TEER monitoring are individually powerful functional readouts, their routine integration within SCO-OoaC devices has not yet been realized. A proof-of-concept for multiplexed biosensing was demonstrated in a multi-organ chip incorporating a PCB-based electrochemical immunobiosensor array capable of simultaneously detecting eight secreted proteins in real time. Extending this approach to SCI-relevant inflammatory cytokines (TNF-α, IL-1β, and IL-6), synaptic vesicle release markers, and metabolic indicators (glucose, lactate, and dissolved O_2_) within a single SCO-OoaC device would substantially advance functional monitoring [160].

### 6.5. Limits of Standard Biological Tests and a Maturation Ceiling

The current standard SCO analytical workflow destroys the 3D spatial information that defines the organoid and is relevant to therapeutic applications. Spatial transcriptomics technologies, such as 10× Visium [162], MERFISH [163], and Slide-seq [164], retain some of this information but remain technically challenging, costly, and incompatible with the longitudinal repeated-sampling designs needed for therapeutic evaluation in organoid systems [43]. These analytical constraints are compounded by a developmental ceiling: current SCO models reconstitute fetal rather than mature spinal cord development and lack the transcriptomic, synaptic, and morphological characteristics of the adult spinal cord. As a result, key chronic SCI pathogenic features, including adult glial scar maturation, long-term Wallerian degeneration, chronic neuropathic pain circuit remodeling, and KCC2-stabilized inhibitory synaptic maturation, remain beyond the developmental capacity of current SCO protocols. The GENtoniK maturation cocktail and extended culture protocols are among the most promising approaches for driving SCO maturation toward adult-equivalent functional properties, but they have not yet been systematically validated in SCO-OoaC systems [63].

## 7. Emerging Solutions and Future Directions

The limitations outlined above define a focused research framework for advancing the SCO-OoaC field. Each bottleneck corresponds to a near-term scientific or technological milestone, often involving an engineering advance or technical strategy that has already shown preliminary feasibility. This section discusses solution-oriented approaches, including immediate developmental priorities, intermediate translational objectives, and long-term goals directed toward clinically integrated SCO-OoaC platforms.

### 7.1. Potential Strategies to Overcome Current Limitations

#### 7.1.1. Immune-Competent SCO-OoaC Platforms

Physiologically relevant integration of immune cells into SCO-OoaC platforms remains a major challenge in developing advanced in vitro spinal cord models. In future studies, timed incorporation of iPSC-derived microglia at organoid days 30–40 could use protocols that preserve homeostatic P2RY12^+^/TMEM119^+^/CX3CR1^+^ identity through sustained fractalkine signaling. This strategy may enable resident immune surveillance capable of transitioning toward a DAM phenotype and activating the NLRP3 inflammasome in response to SCI-conditioned media [165,166,167]. Previously, Ormel et al. demonstrated that incorporating primitive macrophage precursors from the earliest stages of differentiation resulted in spontaneous development of microglia within iPSC-derived brain organoids. This work provides a roadmap for SCO-OoaC systems in which resident microglia maintain authentic transcriptomic identity through self-organization rather than exogenous addition [168].

In the next phase of development, peripheral immune compartments could be created using microfluidic channels containing CCR2+ monocytes, neutrophils, and regulatory T cells under defined cytokine gradients. These compartments would facilitate systematic investigation of peripheral immune-cell diapedesis across the human BSCB in an injury context, directly addressing mechanistic gaps in SCI neuroinflammation modeling [169,170]. Another possibility is integration of OoaC systems with iPSC-based choroid plexus organoids, which would introduce a physiologically important immunological compartment not yet included in current platforms and allow for exploration of its neuroimmune secretory functions. These sequential immune-integration steps could make SCO-OoaC systems useful platforms for studying the spatiotemporal nature of SCI-associated neuroinflammation in a human-specific context [171,172].

#### 7.1.2. Perfusable Vascularized Systems

Because avascular organoids are limited by core necrosis, more sophisticated vascularization strategies are needed. Cakir et al. (2019) showed that ETV2-overexpressing iPSCs mixed with wild-type iPSCs during embryoid body formation spontaneously generated branched vascular networks expressing canonical brain endothelial-cell markers and improved neural viability in organoids larger than 500 μm in diameter [104]. This approach provides a tractable first-generation solution, but avoiding core necrosis will require reliable anastomosis between on-chip vasculature and microfluidic inlets. In addition, two-photon polymerization (2PP) 3D bioprinting can provide sub-100 μm resolution (Table 2), enabling deterministic endothelial-cell seeding of geometrically precise perfusable microvascular scaffolds with channels prespecified to match the distinct capillary architectures of spinal cord gray and white matter. Previously, a study by Quintard et al. reported complete intravascular perfusion of stem cell-derived vascular organoids within a thermoplastic microfluidic platform, resulting in improved endothelial barrier function, vascular maturation, and long-term physiological stability compared with static culture. This study provides a compelling proof of concept for functional on-chip vascularization that may be adaptable to future SCO-OoaC platforms [173].

A fully integrated vascularized–immune–BSCB OoaC platform combining perfusable vasculature, resident iPSC-derived microglia, peripheral immune compartments, and a tricellular neurovascular unit comprising iPSC-derived endothelial cells, pericytes, and astrocytic endfeet under physiological shear stress and continuous TEER monitoring would represent a human cell-based platform capable of simultaneously interrogating hemodynamic, neuroinflammatory, and structural dimensions of SCI-associated BSCB disruption [177,178]. Current animal models and OoC setups do not fully recreate this multicomponent pathophysiology in a human-specific cellular context; therefore, such platforms could provide distinctive value for mechanistic SCI research and therapeutic screening.

### 7.2. Integration of Advanced Biosensing, Multiomics, and Digital Modeling

The convergence of three analytical advances may define the next generation of SCO-OoC platforms by moving beyond current single-parameter endpoint characterization. The main limitation of planar MEA configurations may be addressed by the kirigami electronics (KiriE) platform described by Yang et al. (2024), which consists of flexible SU-8 polymer devices that self-organize into a 3D basket geometry and encapsulate intact organoids [66]. This approach enables long-term, noninvasive volumetric imaging of cortical organoids for 120 days without compromising cytoarchitecture. Integration of KiriE with cell-type-specific GCaMP calcium imaging under two-photon microscopy could provide a combined electrophysiological and optical functional-mapping pipeline, which would be valuable for studying weeks-to-months remyelination and circuit rewiring after SCI [66].

At the biochemical level, PCB-based multiplexed electrochemical immunobiosensor arrays [179], building on the proof-of-concept multi-organ platform of Edington et al., in which up to 10 interconnected microphysiological systems were fluidically coupled for quantitative pharmacokinetic/pharmacodynamic studies [180], may enable simultaneous real-time detection of nine SCI-relevant analytes: TNF-α, IL-1β, IL-6, IL-10, brain-derived neurotrophic factor, NT-3, glucose, lactate, and dissolved O_2_. Detection in OoaC effluent without sample removal could support closed-loop feedback architectures in which real-time biosensor outputs automatically regulate microfluidic flow rates and drug delivery, representing an immediate engineering priority [180].

Longitudinal spatial transcriptomics, such as MERFISH combined with scATAC-seq at defined experimental time points [181], could generate spatiomolecular atlases of SCI propagation and regeneration corridors. These datasets could serve as training corpora for graph neural network and generative artificial intelligence models, enabling patient-specific computational digital twins that prospectively predict therapeutic outcomes from organoid-derived multiomics and electrophysiological data. This approach may represent a major translational step for SCI precision medicine over the next decade [182,183].

### 7.3. Patient-Specific and Precision Medicine

Deriving patient-specific SCO biobanks from iPSCs obtained from SCI patients with defined injury level, severity (American Spinal Injury Association Impairment Scale grades A–D), and genetic background may be one of the most important near-term translational uses of SCO-OoaC systems. Such biobanks could support pharmacogenomic identification of responder and nonresponder drug signatures that cannot be captured in genetically isogenic cell lines or animal models [184]. This concept has been demonstrated in motor neuron disease, where patient-specific SCOs identified therapeutic candidates, including palbociclib for spinal muscular atrophy and DAPT for mitochondrial encephalomyopathy, lactic acidosis, and stroke-like episodes, through organoid-specific mechanisms. A similar approach could be translated to traumatic injury by exposing patient-specific SCOs within OoaC injury platforms to candidate drugs and cell-based therapeutic strategies targeting individual genetic vulnerabilities. In addition, SCO-OoC drug-screening platforms that integrate patient-specific organoids, multiplexed biosensing, automated liquid handling, and MEA electrophysiology could provide multiparameter therapeutic-efficacy profiles that are more relevant to patient-specific response variability than single-endpoint screens [185].

### 7.4. Standardization, Automation, and Manufacturing of SCO-OoaC Platforms

Current SCO-OoaC platforms face reproducibility and scalability challenges that may be addressed through convergent advances in biomaterials, fabrication, and regulatory science. The most immediate standardization step is replacement of Matrigel with chemically defined, tissue-specific dECM hydrogels. Wang et al. (2024) showed that human placenta-derived decellularized ECM can support generation of hiPSC-derived SCOs with greater expression of laminar markers than Matrigel [90], and Wu et al. (2024) reported that decellularized brain ECM hydrogel can produce SCOs with better expression of spinal cord segment-specific markers than Matrigel controls [83]. The next generation of mechanically tunable, non-animal alternatives includes GelMA-dECM composites and methacrylated HA hydrogels with stiffnesses in the 0.5–1 kPa range [186]. On the fabrication side, shifting from manual PDMS soft lithography to automated implementation of injection-molded thermoplastic devices could reduce drug-absorption artifacts and geometric variation while enabling parallel high-throughput, 96-well-compatible automated drug delivery at scale. A commercial example is Emulate’s 2025 AVA platform, which combines automated liquid handling, environmental control, and 96-well-compatible biosensor implementation at scale [144,187].

A multidisciplinary working group led by the NIST published a 2024 framework for the future development and implementation of OoC platform guidelines and standards, complementing the CEN/CENELEC OoC Roadmap developed by 120 European experts and officially presented at EUROoCS 2024. Together, these efforts may establish minimum reporting requirements, interlaboratory validation procedures, and performance benchmarks for OoaC pharmacological data intended to support investigational new drug and new drug application submissions. GMP-certified reference SCO lines are also being established to provide universal positive controls for cross-laboratory validation studies, which are critical for regulatory acceptance [44,188,189].

## 8. Conclusions

This review has described the scientific principles, engineering innovations, therapeutic potential, technological limitations, and translational implications of SCO-OoaC technology as an advance in SCI research and regenerative medicine. In SCO-OoaC systems, human iPSC-derived SCOs provide 3D complexity and self-organization of neural tissue, while bioengineered microdevices provide spatial control, fluidic regulation, and real-time functional monitoring. Together, these features create a more physiologically relevant and human-specific platform for modeling SCI pathophysiology than traditional 2D cultures and may complement animal models [60,190].

Current SCO-OoaC-related platforms and their component technologies have demonstrated the ability to model selected pathological features of SCI, including neuroinflammation, reactive astrogliosis, axonal degeneration, and BSCB dysfunction, although these capabilities have largely been established in separate experimental systems rather than within a single integrated SCO-OoaC platform. Furthermore, several microphysiological platforms have incorporated biosensors, electrophysiological recording systems, and multi-omics analyses, demonstrating the feasibility of longitudinal functional assessment. Their integration into future SCO-OoaC platforms is expected to further enhance disease modeling and therapeutic evaluation [123]. Concurrently, the development of assembloid and immune-competent models has created opportunities to study complex neural circuits, immune–nervous system interactions, and patient-specific disease mechanisms. Moreover, there are three non-replaceable characteristics that set apart the SCO-OoaC platforms from all previous experimental systems, and which together make them irreplaceable in the SCI research landscape. First, SCO-OoaC brings the human-specific molecular authenticity of the human spinal cord, which recreates the precise composition of various CSPG isoforms, NR2B-dominant excitotoxic receptor stoichiometry, and DAM microglial transcriptomic signatures that differ significantly between human and rodent spinal cord tissues, and that define the translatability of pharmacological conclusions to human patients [62,191]. Second, SCO-OoaC provides simultaneous multi-pathology reconstitution with continuous functional monitoring, including quantification of pharmacological rescues by real-time MEA electrophysiology [66], TEER monitoring, and multiplexed biosensing—an integrated device unique in its ability to model and quantify CSPG-mediated axonal growth inhibition, reactive astrogliosis, microglial M1 neuroinflammation, and BSCB disruption concurrently in a single device [66]. Third, SCO-OoaC allows for precision medicine at the tissue level that is patient-specific and can be achieved without performing pharmacologically interrogable human spinal cord tissue from any individual patient, allowing for direct identification of genotype-dependent therapeutic response profiles and prospective stratification of clinical trial populations [43]. Altogether, these advantages are not incremental over current platforms; together they provide a potentially important advance in accessing the human-specific, 3D, multicellular, dynamically monitored, patient-derived spinal cord tissue model necessary to definitively bridge the translational gap in the field of SCI.

Despite these advances, important challenges remain, including limited vascularization, incomplete cellular maturation, interorganoid variability, and the need for standardized and scalable manufacturing methods. Addressing these challenges will be essential for improving reproducibility, predictive validity, and regulatory acceptance. Clinical translation remains at an early stage, but rapid progress in stem cell engineering, biomaterials science, microfluidics, and artificial intelligence-driven analysis is accelerating development in the field [20]. The FDA’s landmark April 2025 guidance, paving the way for using OoC and organoid systems as acceptable NAMs for drug safety assessments, stands as the most momentous regulatory development yet. However, three key challenges still need to be overcome for the data from SCO-OoaC to be routinely included in regulatory submissions. Analytical validity needs to be established by interlaboratory ring studies to prove reproducibility and robustness. Second, biological relevance might be established by retrospective concordance with the well-established human data on drug-responses. Third, GMP-compatible manufacturing standards to produce iPSC-derived cellular components should be developed, to guarantee the consistency and quality of the products. The CEN/CENELEC OoC Standardization Roadmap (2024) and the working group on Microphysiological Systems led by NIST are the most comprehensive current activities for the development of such validation and standardization frameworks [44,188,189]. In addition, there should be a short- and medium-term roadmap for the development of SCO-OoaC platforms. For the near future (1–3 years), it is crucial to standardize cell sources, differentiation protocols, culture conditions, and quality control criteria; to further mature neuronal and glial cells; to include microglia and selected peripheral immune cells; and to develop reproducible injury and BSCB models. In the medium term (3–10 years), the focus should shift towards creating vascularized and immunocompetent organoids that are functional over a longer period of time. Advanced microfluidic systems should provide vascular perfusion, the trafficking of immune cells, and interactions between the neural, vascular, and immune compartments. Concurrently, high-throughput screening, clinical translation, patient-derived cells, automated production and multicenter validation will also be crucial.

In conclusion, human SCO-OoaCs represent a promising platform for SCI modeling, drug screening, and development of regenerative treatments. By integrating human-specific neural tissue with controlled microfluidic environments, these systems may support more personalized therapeutic strategies for patients with spinal cord injury and improve the translation of preclinical findings into clinical applications.

## Figures and Tables

**Table 1:** Conceptual framework of functional modules for SCO-OoaC platforms to model SCI. The summary of each module is from illustrative experimental studies.

Representative Modules	Engineering Design	Commercially Used Equipment Model	SCI-Related Biological Function	Functional Readouts	Evidence Status	Current Limitations	References
**Human iPSC-derived spinal cord organoid platform**	Patterned hPSC-derived SCO cultured in Matrigel, dECM or synthetic hydrogels	Corning^®^ Matrigel [105], CELLINK BIOX bioprinter [105]	Recreates spinal neuronal and glial diversity, regional specification and neuronal circuits formation	HOX domains expression, OLIG2, NKX6.1, ISL1, HB9 (MNX1), ChAT, GFAP, synaptic markers (SYN1, PSD95), spontaneous calcium activity and electrophysiological maturation	Demonstrated in SCO	Immature spinal cord compared to the adult, minimal vascularization, no peripheral immune cells, batch to batch variability	[106,107]
**Microfluidic perfusion system**	Microfluidic device using PDMS with interconnected culture chambers that can be perfused continuously with medium using syringe pumps or hydrostatic pressure	Harvard Apparatus PHD Ultra syringe pump [108], Fluigent MFCS pressure controller [109,110]	Enhances nutrient and oxygen supply, facilitates removal of waste, supports long-term culture of neurons, spatial compartmentalization, induction of localized injury, and delivery of therapeutic agents	Metabolic activity, neurite regeneration, live cell imaging, molecular transport, cell viability, drug response	Reported in neural microfluidic system, and emerging SCO platform	Device fabrication complexity, flow optimization, bubble formation, limited standardization, and incomplete recapitulation of the in vivo spinal cord microenvironment	[111,112]
**Axon-guidance/compartmentalized injury module**	Microchannels that permit axonal extension while restricting cell-body migration	Xona Microfluidics (SND150) [113], and slide mounted tool AXIS™ Axon Isolation Device (Merck Millipore) [114]	Enables spatial separation of neuronal somata and distal axons, mimicking tract-specific axonal injury and neuron–glia interaction relevant to SCI	Axonal outgrowth, degeneration, regeneration, transport, axotomy response, and neurotrophic-factor responsiveness	Demonstrated in microfluidic neuronal and emerging in human spinal cord organoid-on-a-chip system	Primarily models isolated axonal injury rather than complex spinal cord tissue; lacks vascular, immune and multicellular interactions in most current platforms	[111,112,115,116]
**Blood–spinal cord barrier/neurovascular module**	Microfluidic neurovascular platform including endothelial cells cultured under dynamic flow with astrocytes and pericytes in adjacent compartments separated by a porous membrane or ECM hydrogel	iBMEC, Human organ mimic device (Emulate Organ-Chips [117], Mimetas OrganoPlate [118], SynVivo chips/SynBBB) [119]	Recapitulating BSCB integrity, endothelial–glial crosstalk, vascular permeability, inflammatory responses, and drug transport relevant to SCI	TEER, permeability assays, tight-junction proteins, inflammatory cytokine transport, and drug penetration	Reported in BBB/BSCB-on-chip platforms; and also emerging in SCI-specific BSCB models	Limited availability of human spinal cord-specific endothelial cells; incomplete immune integration; few fully integrated SCO-BSCB platforms	[117]
**Electrophysiological monitoring module**	Integrated suspended mesh MEAs, calcium imaging, optogenetics, and emerging 3D flexible electronics for longitudinal monitoring of neural organoids	Measure cellular behavior and electrical activity (Axion BioSystems Maestro Pro, Multi-Channel Systems MEA2100) [120]	Evaluates functional maturation of spinal neural circuits and quantifies electrophysiologic dysfunction and recovery after injury and therapeutic interventions	Spike rate, burst frequency, network synchrony, and responses to drugs or injury stimuli	Demonstrated in neural organoids; emerging in SCO-OoaC platforms	Planar MEAs cannot record deep neurons; calcium imaging may cause photobleaching and phototoxicity during chronic imaging; optogenetics require genetic modification	[65,66]
**Secondary injury-mimetic module**	Induced delivery of glutamate, H_2_O_2_, TNF-α, IL-1β, LPS, hypoxia, or OGD in microfluidic channels or culture media in a localized manner	N/A	Represents important secondary SCI pathophysiological features such as excitotoxicity, oxidative stress, neuroinflammation, mitochondrial dysfunction, apoptosis and disruption of BSCB	ROS levels, mitochondrial dysfunction, caspase activation, axonal degeneration, and cell death	Demonstrated in neural organoids and emerging in SCO-OoaC systems	Acute chemical injury does not fully reproduce mechanical contusion or compression SCI	[51,121]
**Drug-screening/therapeutic testing module**	Parallelized chip formats allowing for controlled exposure to small molecules, biologics, EVs, gene vectors, or biomaterials	Mimetas OrganoPlate^®^ [119], Emulate Organ-Chip, CN Bio PhysioMimix	Enables human-relevant testing of neuroprotective, regenerative, and barrier-modulating therapies	Axon regeneration, neuronal survival, inflammatory suppression, barrier recovery, and electrophysiological rescue	Demonstrated in emerging SCO-OoaC system	Throughput, standardization, cost, and regulatory validation remain major barriers	[122,123,124]

**Abbreviations: **BBB, blood–brain barrier; BSCB, blood–spinal cord barrier; dECM, decellularized extracellular matrix; EV, extracellular vesicle; iPSC, induced pluripotent stem cell; MEA, microelectrode array; OoaC, organoid-on-a-chip; ROS, reactive oxygen species; SCI, spinal cord injury; SCO, spinal cord organoid; TEER, transendothelial electrical resistance; N/A, Not Available.

**Table 2:** Comparison of 2PP bioprinting and stem cell self-vascularization strategies for SCO-OoaC platforms.

Feature	2PP Bioprinting	Stem Cell Self-Vascularization
Principle	Laser-fabricated vascular channels	Endothelial self-assembly
Vessel formation	Immediate	10–21 days
Equipment cost	Very high	Moderate
Biological fidelity	Moderate	High
Reproducibility	High	Moderate
Scalability	Limited	Better
Current limitation	Expensive instrumentation	Long maturation time
References	[174,175]	[176]

## Data Availability

No new data were created or analyzed in this study. Data sharing is not applicable to this article.

## Abbreviations

The following abbreviations are used in this manuscript:

BSCB	Blood–spinal cord barrier
BMP	Bone morphogenetic protein
CSPG	Chondroitin sulfate proteoglycan
dECM	Decellularized extracellular matrix
ECM	Extracellular matrix
EV	Extracellular vesicle
FDA	Food and Drug Administration
GelMA	Gelatin methacryloyl
GMP	Good manufacturing practices
HA	Hyaluronic acid
hiPSCs	Human induced pluripotent stem cells
hESCs	Human embryonic stem cells
MEA	Microelectrode array
MMP	Matrix Metalloproteinases
NIST	National Institute of Standards and Technology
OoaC	Organoid-on-a-chip
OoC	Organ-on-a-chip
SCO	Spinal cord organoid
SCI	Spinal cord injury
SCO-OoaC	Spinal cord organoid-on-a-chip
SHH	Sonic hedgehog
TEER	Transepithelial/Transendothelial electrical resistance
VEGF	Vascular endothelial growth factor
WHO	World Health Organization
